# Exploring the mechanism of Yin Huo decoction in PCPA-induced depression mice: a metabolomics and network pharmacology approach

**DOI:** 10.3389/fnmol.2025.1725806

**Published:** 2026-04-02

**Authors:** Yu Zhou, Lu Jia Liu, Yue Zhang, Wen Wen Wang, Dan Hong Xu

**Affiliations:** 1 Wuxi Higher Health Vocational Technology School, Wuxi, Jiangsu, China; 2 College of Pharmacy, Heilongjiang University of Chinese Medicine, Harbin, China; 3 Wuxi Affiliated Hospital of Nanjing University of Chinese Medicine, Wuxi, Jiangsu, China

**Keywords:** depression, mechanism of action, metabolomics, network pharmacology, Yin Huo decoction

## Abstract

**Introduction:**

Depression is a prevalent neuropsychiatric disorder, and traditional Chinese medicine formulations such as Yin Huo Decoction (YHD) have shown potential antidepressant effects, yet their underlying mechanisms remain incompletely elucidated. This study aimed to investigate the therapeutic effects and molecular mechanisms of YHD in a PCPA-induced depression model in mice.

**Methods:**

PCPA-induced depressive-like mice were treated with YHD, and changes in body weight, sucrose preference, and behavioral performance in the forced swim and tail suspension tests were assessed. Hippocampal neuron structure and Nissl body integrity were examined, and brain serotonin (5-HT) levels were quantified. Liquid Chromatograph Mass Spectrometer (LC–MS)-based metabolomic profiling was performed on serum, urine, and brain tissue to identify metabolic disturbances, while network pharmacology analysis was used to explore the intersection of YHD targets and depression-related pathways. Pathway enrichment analysis was conducted to clarify key regulatory pathways.

**Results:**

YHD treatment significantly improved body weight, sucrose preference, and depressive-like behaviors in PCPA-induced mice, and preserved hippocampal neuron structure and Nissl body integrity—effects comparable to fluoxetine. YHD also restored reduced brain 5-HT levels in PCPA model mice. Metabolomic analysis revealed distinct metabolic perturbations in the PCPA model (e.g., in tryptophan and riboflavin metabolism), which were largely reversed by YHD. Network pharmacology identified 156 intersecting targets between YHD and depression-related pathways, primarily involved in neuroactive ligand-receptor interactions, dopaminergic synapses, and inflammatory processes (e.g., TNF signaling and cytokine production). Key targets including AKT1, TNF, IL-6, and EGFR were identified as central to YHD’s action.

**Discussion:**

YHD alleviates PCPA-induced depression-like behaviors in mice by modulating 5-HT levels, correcting metabolic imbalances in tryptophan and riboflavin pathways, and regulating neuroinflammation, neurotransmitter systems, and cellular signaling via targets such as AKT1 and TNF. These findings provide a comprehensive mechanistic understanding of YHD’s antidepressant effects, supporting its potential as a therapeutic agent for depression.

## 1 Introduction

Depression is a common mental disorder characterized by a persistent low mood and/or anhedonia. The pathogenesis of depression is mainly linked to a depletion of monoamine neurotransmitters, hyperactivity of the hypothalamic–pituitary–adrenal (HPA) axis, and the overexpression of inflammatory cytokines (Miller and Campo, 2021; Jesulola et al., 2018; Zhou et al., 2022; Halaris, 2019). Clinical studies have shown that patients with depression display abnormal metabolism of the neurotransmitter 5-hydroxytryptamine (5-HT) and its related metabolites (Li et al., 2015). A reduction in 5-HT levels contributes to depressive symptoms by disrupting circadian rhythm homeostasis (Crouse et al., 2021; Bunney et al., 2015), highlighting the critical role of 5-HT in depression development (Marszalek-Grabska et al., 2021). Currently, the main pharmacological treatments for depression include selective serotonin reuptake inhibitors (SSRIs) and monoamine oxidase inhibitors (MAOIs). The commonly used first-line drugs include fluoxetine, citalopram and amitrityline, etc. However, such drugs can cause gastrointestinal reactions, weight gain, insomnia, anxiety, and even sexual dysfunction. Yin Huo Decoction (YHD), as an extract of natural drugs, has multi-component compound compatibility and multi-target synergy, with few side effects and no risk of drug resistance, and can enhance the body’s self-healing ability to a certain extent. It can often achieve the effect of “curing the root cause” (Li et al., 2020). As a result, there is growing interest in identifying alternative therapeutic options that offer improved safety and efficacy profiles. Traditional Chinese Medicine (TCM), known for its multi-component and multi-target properties, has shown promising potential in treating depression, offering both efficacy and low toxicity (Xu et al., 2022).

From a TCM perspective, depression is believed to result from an imbalance between Yin and Yang, a deficiency in Yang qi, and subsequent disruptions in mental activity. Specifically, kidney Yin deficiency and heart fire (inflammation) are considered the primary pathological factors, with kidney tonification being a key therapeutic approach in TCM (Kunlingzi and Lisheng, 2020). YHD is a classical TCM formula used to tonify the kidneys. It originates from the Qing Dynasty’s Syndrome Differentiation and Qiwen’ and comprises Rehmannia glutinosa (prepared root), Morinda officinalis, *Ophiopogon japonicus*, Schisandra chinensis, and Poria cocos (Ying et al., 2015). Previous studies have shown that YHD can alleviate perimenopausal depression in rats (Dongxue et al., 2023), as well as depression- and anxiety-like behaviors in both long-term and short-term ovariectomized mice (Shurong et al., 2023). Despite these promising results, existing research on YHD remains limited, and more comprehensive studies are needed to fully elucidate its therapeutic potential and underlying mechanisms.

P-chlorophenylalanine (PCPA) is a model of depression constructed by inhibiting the activity of tryptophan hydroxylase (TPH) and thereby reducing serotonin levels (Daut and Fonken, 2019; Zhikang and Fei, 2023). Therefore, we evaluated the antidepressant effect of YHD under this model and explored its molecular mechanism. A mouse model of depression was established by intraperitoneal injection of PCPA, a selective inhibitor of tryptophan hydroxylase, followed by YHD treatment via oral gavage. Behavioral assays, histological analyses, and enzyme-linked immunosorbent assays (ELISA) were perdormed to assess depression-like behaviors and 5-HT levels in brain tissue, thereby evaluating YHD’s therapeutic efficacy.

Furthermore, we also used high-throughput metabolomics was used to identify differential metabolites in serum, urine, and brain tissue, thereby elucidating the metabolic profile associated with YHD treatment. Finally, network pharmacology was employed to explore the molecular mechanisms underlying YHD’s antidepressant effects, and to provide a certain basis for the future research on the core targets and active ingredients of YHD in the treatment of depression.

## 2 Materials and methods

### 2.1 Animals

Nine-week-old male KM mice (25 ± 2 g) were obtained from the Faculty of Laboratory Animal Science, Harbin Medical University. The mice were acclimated for 1 week prior to the start of the experiments. All animals were housed under specific-pathogen-free (SPF) conditions, maintained at a controlled temperature of 24 ± 0.5 °C and relative humidity of 55 ± 5%. The mice were provided ad libitum access to standard chow and water. In accordance with the ARRIVE 2.0 standard, we have supplemented the sample size of each experimental group in the original text and clarified the relevant matters regarding the blind method. All experimental procedures were conducted in accordance with institutional guidelines and relevant regulations governing the care and use of laboratory animals. Approval number: No.2023052601.

We selected 9-week-old male KM mice due to their well-established use in depression models and their stable genetic background, which allows for more consistent and reproducible results. These mice are mature, with stable hormonal levels that minimize potential confounding factors related to sex hormones. However, we acknowledge that the generalizability of our findings may be limited to male KM mice, and future studies should consider the potential differences in antidepressant efficacy between sexes and genetic strains.

### 2.2 Experimental groups and treatment

The PCPA model is a well-recognized and widely used model in rodents to induce depressive-like behaviors by reducing serotonin synthesis. As a selective and irreversible inhibitor of TPH, PCPA inhibits TPH activity, thereby reducing serotonin synthesis, which mimics the serotonin deficiency observed in depression.

The specific operation for constructing the depression model with PCPA is as follows: PCPA (D831376; Macklin, Shanghai, China) was dissolved in 0.9% physiological saline as the solvent and thoroughly mixed to prepare a suspension at a concentration of 45 mg/mL. The pH of this solution was approximately 5.5 as detected. In subsequent modeling experiments, the suspension was intraperitoneally injected into mice at a dose of 450 mg/kg once daily for 4 consecutive days. This protocol is consistent with the classic approach in previous studies and can significantly lower serotonin levels and induce depressive-like behaviors (Kukuia et al., 2022).

To evaluate the antidepressant effects of YHD, mice were treated with the decoction following model induction. YHD is composed of Rehmanniahg nbAQ glutinosa (Shu Dihuang), Morinda officinalis (Ba Jitian), *Ophiopogon japonicus* (Mai Dong), Poria cocos (Fu Ling), and Schisandra chinensis (Wu Weizi), all sourced from Beijing Tongrentang Pharmaceutical Co. (Harbin, China). Mice were administered YHD at a dose of 4.05 g/kg via intragastric gavage (i.g.) once daily for 7 days.

Sixty-four KM mice were housed under SPF conditions (temperature: 24 ± 2 °C, humidity: 55 ± 10%) with a 12:12 light/dark cycle. The mice had ad libitum access to commercial SPF chow and autoclaved water. Following a one-week acclimatization period, they were randomly assigned to four groups: Control group (C): healthy mice receiving purified water (i.g.) for 7 days. Model group (M): PCPA-treated mice receiving purified water (i.g.) for 7 days. YHD group (Y): PCPA-treated mice receiving YHD (4.05 g/kg, i.g.) once daily for 7 days. Fluoxetine group (F): PCPA-treated mice receiving fluoxetine (2.6 mg/kg; 5558A, Eli Lilly and Company) via i.g. administration once daily for 7 days.

**Figure 1:**
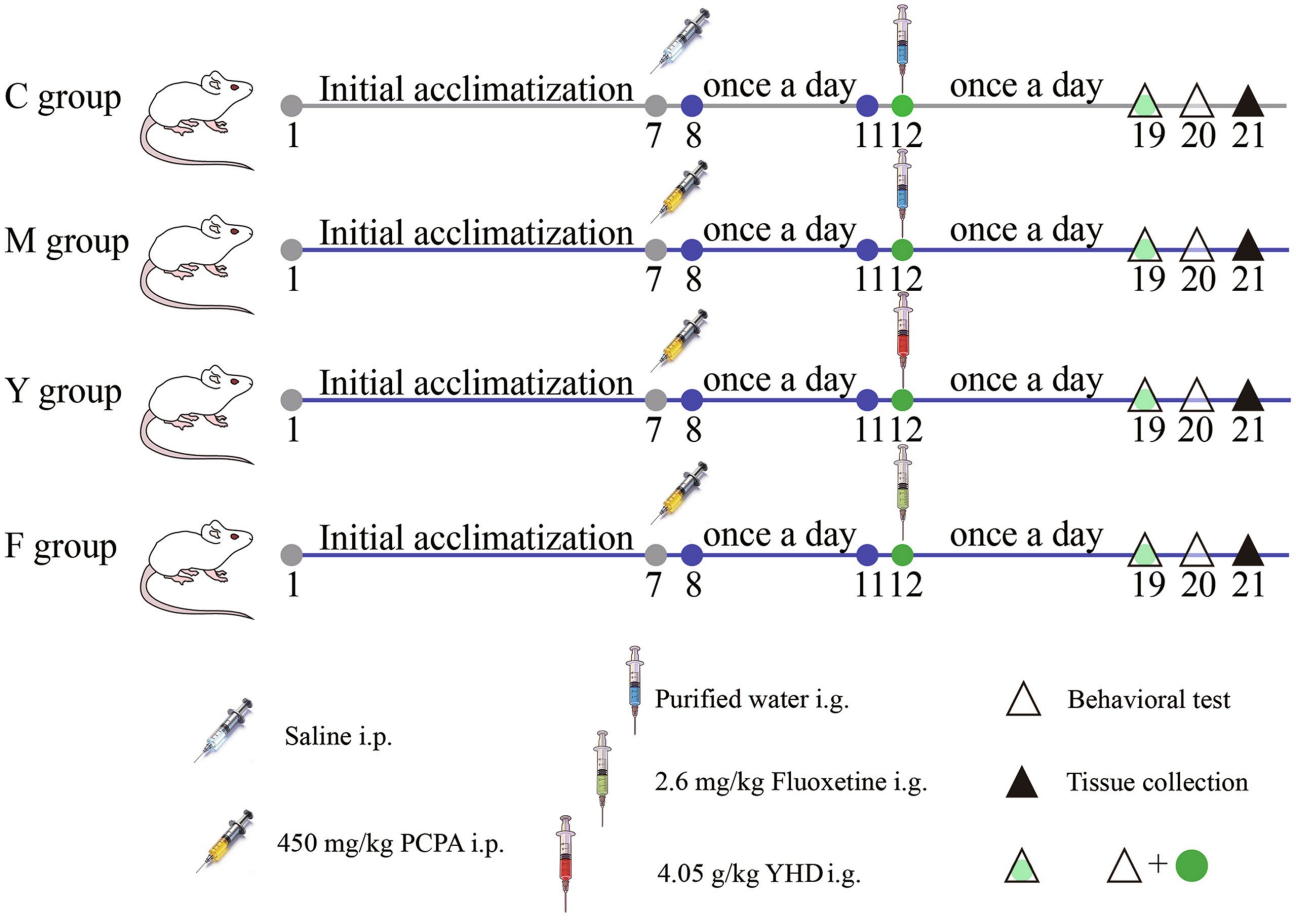
Schematic diagram showing the detail of PCPA stimulation and drug treatment in PCPA-induced depression mice model.

Twenty-four hours after the final PCPA injection, YHD and fluoxetine treatments were initiated in the respective groups. Control and model groups received an equivalent volume of purified water. Following the final dose, behavioral tests were conducted over 2 days. Then, under deep anesthesia with isoflurane (2 ~ 3%; S190815, Yuyuan, Shanghai, China), mice were transcardially perfused with ice-cold sterilized saline, and then were collected for futher analysis. The experimental design is illustrated in Figure 1.

### 2.3 Behavioral tests

#### 2.3.1 Sucrose preference test (SPT)

The Sucrose Preference Test (SPT) was used to assess anhedonia, a core symptom of depression. Prior to testing, mice were habituated to a 1% sucrose solution. On the first day of habituation, mice were presented with two identical bottles containing 1% sucrose solution. After 24 h, one bottle was replaced with purified water. To avoid side preference, the positions of the bottles were alternated every 6 h. Mice were deprived of water for 24 h before the test. On the testing day, each mouse was provided with one bottle of 1% sucrose solution and one bottle of purified water. The positions of the bottles were swapped every hour to minimize positional bias. Finally, the consumption of sucrose after 2 h was recorded and calculated by the following formula: SPT (%) = [Sucrose consumption / (Sucrose consumption + Water consumption)] × 100.

#### 2.3.2 Forced swimming test (FST)

The Forced Swimming Test (FST) was employed to evaluate depression-like behavior, specifically behavioral despair. Each mouse was placed individually in a transparent Plexiglas cylinder (40 cm height × 20 cm diameter) filled with water to a depth of 25 ± 3 cm, maintained at 24 ± 1 °C. Mice were observed for 6 min, and the duration of immobility during the final 4 min was recorded. Immobility was defined as the absence of active movements, with the mouse floating passively or making only minimal movements to keep its head above water.

#### 2.3.3 Tail suspension test (TST)

The Tail Suspension Test (TST) was conducted to assess behavioral despair in mice. Following the final intragastric administration, mice were individually suspended by the tail using adhesive tape, positioned 1 cm from the tail tip. The mice were suspended at a height of 25 cm above the surface for 6 min. The duration of immobility during the last 4 min was recorded. Mice were considered immobile when they remained completely motionless and passive.

#### 2.3.4 Open field test (OFT)

The Open Field Test (OFT) was utilized to assess anxiety-like behavior and general locomotor activity. The open field apparatus consisted of a square arena (100 cm × 100 cm × 40 cm) with a black floor divided into nine equal squares. Following the last administration, each mouse was placed in the center of the arena and allowed to explore freely for 5 min under dim lighting and a quiet environment. Behavioral parameters recorded included the number of grid crossings (locomotor activity), entries into the central area (anxiety-related behavior), instances of rearing (standing on hind legs), and grooming behaviors. The apparatus was thoroughly cleaned with 75% ethanol between trials to eliminate odor cues.

### 2.4 Sample collection and tissue preparation

After the completion of behavioral experiments, blood samples were collected via orbital bleeding. Following this, a thoracotomy was performed to expose the heart, and a small incision was made in the right atrium. Three mice from each group were selected for perfusion: an intragastric administration needle was inserted into the apex of the heart, and perfusion was conducted until both the liver and the perfusate appeared pale, indicating complete circulation clearance.

Immediately post-decapitation, the brains were carefully dissected on an ice-cooled dish. The olfactory bulbs and cerebellum were removed, and the brains were bisected into left and right hemispheres using a sterile blade. The hemispheres were fixed in 4% paraformaldehyde for 48 h. Following fixation, the brain tissues were embedded in paraffin for subsequent Hematoxylin–Eosin (HE) and Nissl staining.

The remaining 13 mice were decapitated without perfusion, and their brains were similarly dissected into hemispheres. These samples were rapidly frozen in liquid nitrogen and stored at −80 °C for enzyme-linked immunosorbent assay (ELISA) and metabolomics analyses.

### 2.5 Hematoxylin–eosin (HE) staining

HE staining was performed following standard protocols. Briefly, fixed brain tissues were dehydrated through a graded ethanol concentrations and then embedded in paraffin at 60 °C. Paraffin-embedded tissues were sectioned, deparaffinized, and rehydrated. Sections were stained with hematoxylin and eosin solutions (DH0006, Beyotime Biotechnology) for 5 min. Following staining, the sections were dehydrated, cleared, and mounted with coverslips. Histopathological changes in the brain tissue were observed under a light microscope.

### 2.6 Nissl staining

Nissl bodies, which serve as markers of neuronal health by indicating the synthesis of structural proteins necessary for cellular function, diminish or disappear in response to neuronal damage. After undergoing the same dehydration, clearing, infiltration, embedding, and sectioning procedures as in HE staining, brain sections were stained with 0.1% toluidine blue solution (C00117, Beyotime Biotechnology). Following staining, sections were dehydrated, cleared, and sealed. Neuronal morphology and the presence of Nissl bodies were examined under a light microscope.

### 2.7 Enzyme-linked immunosorbent assay (ELISA)

The 5-HT quantification via ELISA, total protein was extracted from homogenized tissue of the left hemisphere of the mouse brain. The tissue was homogenized in 200 μL RIPA buffer (P0013C, Beyotime Biotechnology, Shanghai, China) supplemented with 2 μL phenylmethanesulfonyl fluoride (PMSF; ST506, Beyotime, Shanghai, China) and 4 μL phosphatase inhibitors (P1081, Beyotime, Shanghai, China). The homogenates were centrifuged at 17,949 × g for 5 min at 4 °C, and the supernatants were collected. Protein concentrations were quantified using a BCA protein assay kit (BL521A, Biosharp, Hefei, China). The 5-hydroxytryptamine (5-HT) levels in brain tissue were measured according to the manufacturer’s instructions using commercial ELISA kits (H104-1-1, Nanjing Jiancheng Bioengineering Institute).

### 2.8 Untargeted metabolomics analysis using HPLC–QTOF-MS

An ExionLC™ AD system coupled with a TripleTOF™ 5,600^+^ mass spectrometer (SCIEX) was employed to perform untargeted metabolomics analysis.

#### 2.8.1 Chromatography detection conditions

Utilizing a column, ACQUITY UPLC HSS T3 (100 mm × 2.1 mm, 1.8 μm), the experiment conducted at 40 °C, with an injection volume of 5 μL and a detection time of 23 min. The mobile phase consisted of 0.1% formic acid aqueous solution (phase A) and 0.1% formic acid-acetonitrile (phase B). The elution program is presented in Table 1.

**Table 1:** Gradient elution procedure.

Time (min)	Flow (mL/min)	A (%)	B (%)
0	0.3	95	5
2	0.3	95	5
6	0.3	70	30
7	0.3	70	30
10	0.3	40	60
11.5	0.3	40	60
13	0.3	20	80
13.5	0.3	20	80
16	0.3	10	90
17	0.3	10	90
17.5	0.3	0	100
20	0.3	0	100
20.5	0.3	95	5
23	0.3	95	5

#### 2.8.2 Mass spectrometry detection conditions

Positive and negative ion detection modes were used, with the first and second mass spectrometry scan ranges set at 100–1200 m/z and 50–1,200 m/z, respectively. Nitrogen gas was used for all gas paths. For specific parameters, please refer to Table 2.

**Table 2:** Mass spectrometry conditions.

Parameter	LC–MS	LC–MS/ MS
Scan Type	TOF	TOF
Ion Spray Voltage Floating (ISVF)	±5,500 V	±5,500 V
Source Gas 1 (GS1)	60 psi	60 psi
Source Gas 2 (GS2)	60 psi	60 psi
Curtain Gas (CUR)	35 psi	35 psi
Temperature (TEM)	550 °C	550 °C
Declustering Potential (DP)	100 V	100 V
Collision Energy (CE)	±10 V	±40 V
Collision Energy Spread (CES)	—	20 V

#### 2.8.3 Serum sample collection and processing

Allow the blood to stand for 30 min. Centrifuge at 4000 rpm for 15 min at 4 °C to collect the supernatant. Precipitate proteins with methanol. Centrifuge at 13000 rpm for 15 min at 4 °C, then evaporate the supernatant using nitroge. Resuspend in methanol and vortex to mix thoroughly. Centrifuge the blood homogenate at 13000 rpm for 10 min at 4 °C. Filter the supernatant through a 0.22 μm membrane filter, then inject into the machine. Quality control (QC) samples were prepared by mixing 10 μL of each sample.

#### 2.8.4 Urine sample collection and preparation

Collect mouse urine, mix with distilled water at a 1:2 ratio (total volume 300 μL), vortex for 10 s to obtain a uniform urine suspension. Subsequent methods are equivalent to blood homogenization. Prepare QC samples as described previously.

#### 2.8.5 Brain tissue sample collection and preparation

Brain tissue homogenates were prepared by homogenizing the right hemisphere of the mouse brain in a 1:1 methanol–water solution. Subsequent methods are equivalent to blood homogenization. Prepare QC samples as described above.

### 2.9 Data processing

An untargeted metabolomics approach was utilized to characterize differential metabolites in serum, urine, and brain tissue. Data processing and metabolite identification were conducted using MS-DIAL software to integrate comprehensive metabolic profiles. For targeted metabolomics, data were processed using SCIEX OS software. Quantitative results were derived by applying the peak area ratios (analyte/internal standard) to corresponding standard curves to calculate the concentration of each metabolite.

Metabolomics data were processed using the following methods: The raw data were converted to the ABF format using the Analysis Base File Converter, followed by preprocessing with MSDIAL software. The metabolite identification library was based on LC–MS/MS data in both Positive and Negative Ion Modes^1^. Multivariate statistical analysis was conducted using SIMCA software, which included unsupervised Principal Component Analysis (PCA), Partial Least Squares Discriminant Analysis (PLS-DA), and Orthogonal PLS-DA (OPLS-DA). Differential metabolites were analyzed using MetaboAnalyst^2^, allowing for the identification of potential metabolic pathways. The trends of differential metabolites across various metabolic pathways were compiled to construct a metabolic disturbance map.

### 2.10 Network pharmacology analysis

#### 2.10.1 Identification of YHD targets and depression-associated genes

TCMSP, SwissTargetPrediction and PharmMapper databases were used for prediction and analysis to obtain the potential targets of YHD. In addition, targets associated with depression were collected from DisGeNET, OMIM, and GeneCards databases. The target data of YHD and depression were imported into the Venn graph making website for intersection target analysis to provide further research.

#### 2.10.2 Protein–protein interaction (PPI) network and core target analysis

The common targets of YHD and depression obtained from network pharmacology analysis were imported into the STRING database for PPI network construction, and disconnected nodes were excluded to enhance the robustness of the network. The generated interaction files were imported into Cytoscape software to visualize the PPI network.

#### 2.10.3 GO enrichment and KEGG pathway analysis

Gene Ontology (GO) enrichment analysis and Kyoto Encyclopedia of Genes and Genomes (KEGG) pathway enrichment were conducted using the Metascape platform. The top five terms from biological processes (BP), cellular components (CC), and molecular functions (MF) were selected based on *p*-values for visualization.

#### 2.10.4 Construction of the “YHD components–depression–target–pathway” network

Active YHD components capable of crossing the blood–brain barrier, together with the intersecting targets and KEGG-enriched pathways, were integrated into Cytoscape to construct a comprehensive network illustrating the relationship between YHD components, depression-related targets, and pathways.

### 2.11 Statistical analysis

Statistical analyses were performed using SPSS 21.0 software. Data are presented as mean ± SD for normally distributed variables. Comparisons across multiple groups were analyzed using one-way ANOVA, followed by Tukey’s Honest Significant Difference (HSD) test for pairwise comparisons. Statistical significance was defined as *p* < 0.05 (significant), *p* < 0.01 (highly significant), and *p* < 0.001 (extremely significant).

## 3 Results

### 3.1 Effects of YHD on body weight and depressive-like behaviors in PCPA model mice

**Figure 2:**
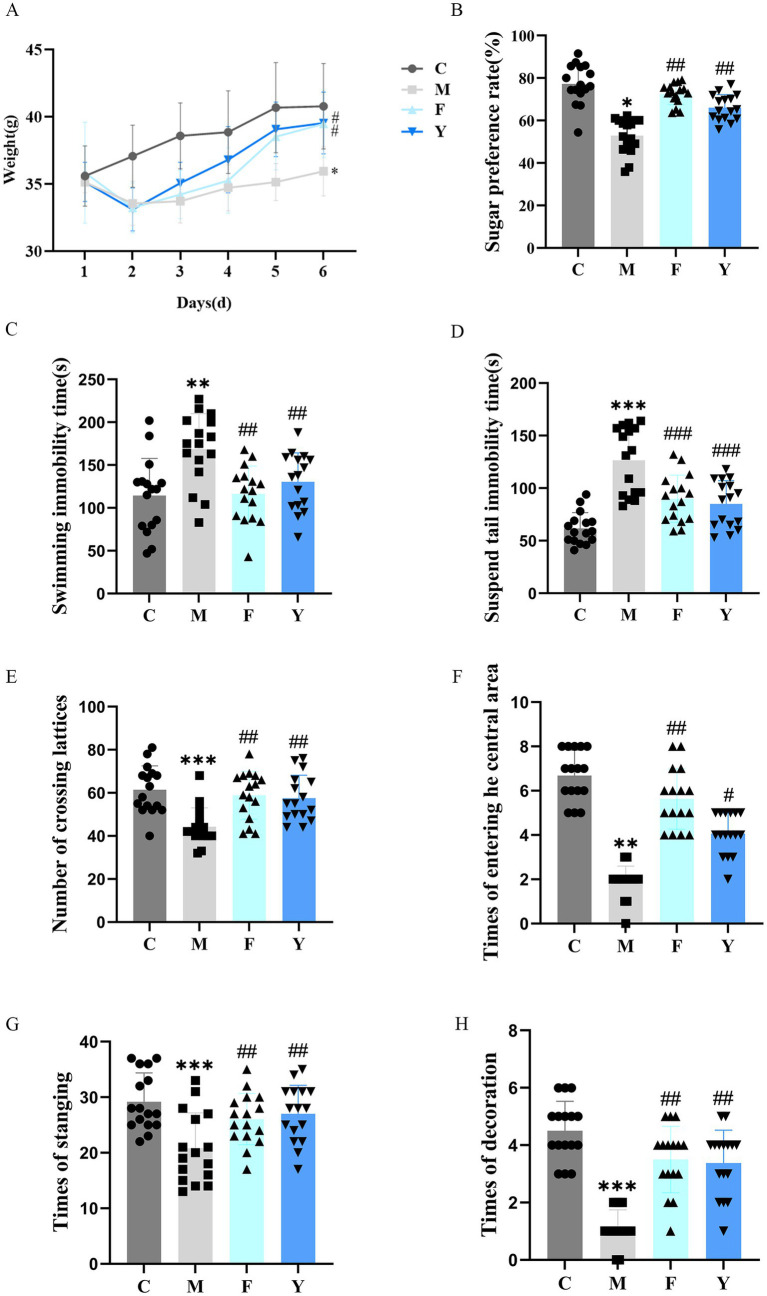
Effects of YHD on the body weight and behavioral test in each group of mice. **(A)** Changes in body weight of mice within 6 days after modeling (*n* = 16 per group). **(B)** Effect of YHD on sugar water preference rate in PCPA model mice (*n* = 16 per group). **(C)** Effect of YHD on the immobility time of swimming in PCPA model mice (*n* = 16 per group). **(D)** The effect of YHD on the immobility time of tail suspension in PCPA model mice (*n* = 16 per group). **(E–H)** The effect of YHD on the open field experiment of PCPA model mice (*n* = 16 per group). ^*^ *p <* 0.05, ^**^ *p <* 0.01, ^***^p < 0.001 vs. control group; ^#^ *p <* 0.05, ^##^ *p <* 0.01 vs. model group.

To evaluate the effects of YHD on PCPA-induced depression model mice, we first monitored body weight changes for 6 days post-modeling. The body weight of the M group was significantly lower than that of the C group (*p* < 0.05). Post-treatment with YHD or fluoxetine significantly increased the body weight of PCPA model mice (*p* < 0.05) (Figure 2A).

In the sucrose preference test, the M group showed a significant reduction in sucrose preference compared to the C group (*p* < 0.01), while YHD (Y) and fluoxetine (F) treatments significantly improved preference (*p* < 0.01) (Figure 2B). In the forced swim test, the M group exhibited increased immobility time compared to the C group (*p* < 0.01), which was reduced by both YHD and fluoxetine treatment (*p* < 0.01) (Figure 2C). Similarly, in the tail suspension test, the M group had significantly increased immobility time (*p* < 0.001), which was alleviated by YHD and fluoxetine (*p* < 0.001) (Figure 2D). In the open field test, the M group displayed reduced movement and exploratory behaviors, with fewer grid crossings, central area entries, rearing, and grooming events (*p* < 0.01, *p* < 0.001). YHD and fluoxetine treatments significantly improved these behaviors (*p* < 0.05, *p* < 0.01) (Figures 2E–H).

These results confirm the successful establishment of the PCPA-induced depression model and demonstrate that YHD effectively alleviates PCPA-induced weight loss and motor dysfunction. Furthermore, YHD was consistent with fluoxetine results in improving anhedonia, hopelessness, and anxiety.

### 3.2 Histological effects of YHD on hippocampal neurons, Nissl bodies, and brain 5-HT content in PCPA model mice

**Figure 3:**
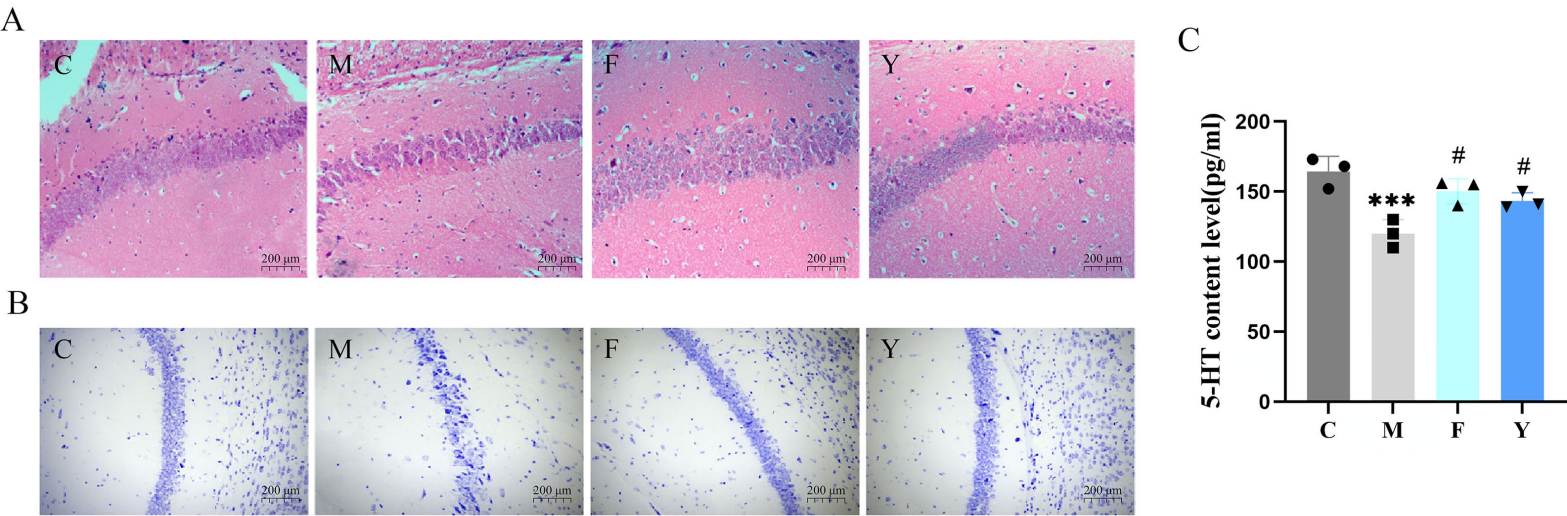
Results of histomorphological observation. **(A)** Effect of YHD on hippocampal neurons in PCPA model mice at 10 × magnification (*n* = 3 per group). **(B)** The effect of YHD on the Nissl body in the hippocampus of PCPA model mice at 10 × magnification (*n* = 3 per group). **(C)** Effects of YHD on 5-HT content in PCPA mice (*n* = 3 per group). ^*^p < 0.05, ^**^ *p* < 0.01 ^***^p < 0.001 vs. control group; ^#^ *p* < 0.05, ^##^ *p* < 0.01 vs. model group.

HE and Nissl staining were used to assess hippocampal neuron morphology in PCPA mice, evaluating the effects of YHD treatment. HE results showed that hippocampal neurons in the control (C) group exhibited a well-preserved cell structure, compact arrangement, and distinct nucleoli. In contrast, the M group showed significant neuronal degeneration, characterized by scattered and disorganized neurons with unclear structures, pyknosis of the nuclei, and a reduced number of cells. However, in the F and Y groups, the structure of hippocampal neurons was relatively intact, with a more uniform distribution and an increased number of cells (Figure 3A). Disruption of Nissl substance integrity reflects impaired neuronal function and is one of the key pathological features of depression (Duman and Monteggia, 2006; Peng et al., 2015).

Similarly, Nissl bodies were abundant in the hippocampal neurons of the C group, whereas in the M group, the Nissl bodies were lighter and fewer in number. In contrast, the F and Y groups displayed darker, more abundant Nissl bodies, along with a significant increase in the number of cells compared to the M group (Figure 3B). These findings suggest that YHD improves the morphology and number of hippocampal neurons and Nissl bodies in PCPA model mice.

5-HT, a key neurotransmitter involved in depression, has been shown to be significantly reduced in PCPA-induced depression models (Gumuslu et al., 2013). The brain tissue content of 5-HT was measured using ELISA. As reported previously, the 5-HT content in the M group was markedly decreased compared to the C group (*p* < 0.001). Both YHD and fluoxetine treatments reversed the reduction in 5-HT levels observed in the M group (*p* < 0.01, *p* < 0.05) (Figure 3C). This indicates that YHD can increase the 5-HT content in the brain of PCPA model mice.

### 3.3 Effects of YHD on serum metabolic profile and differential metabolites in PCPA model mice

**Figure 4:**
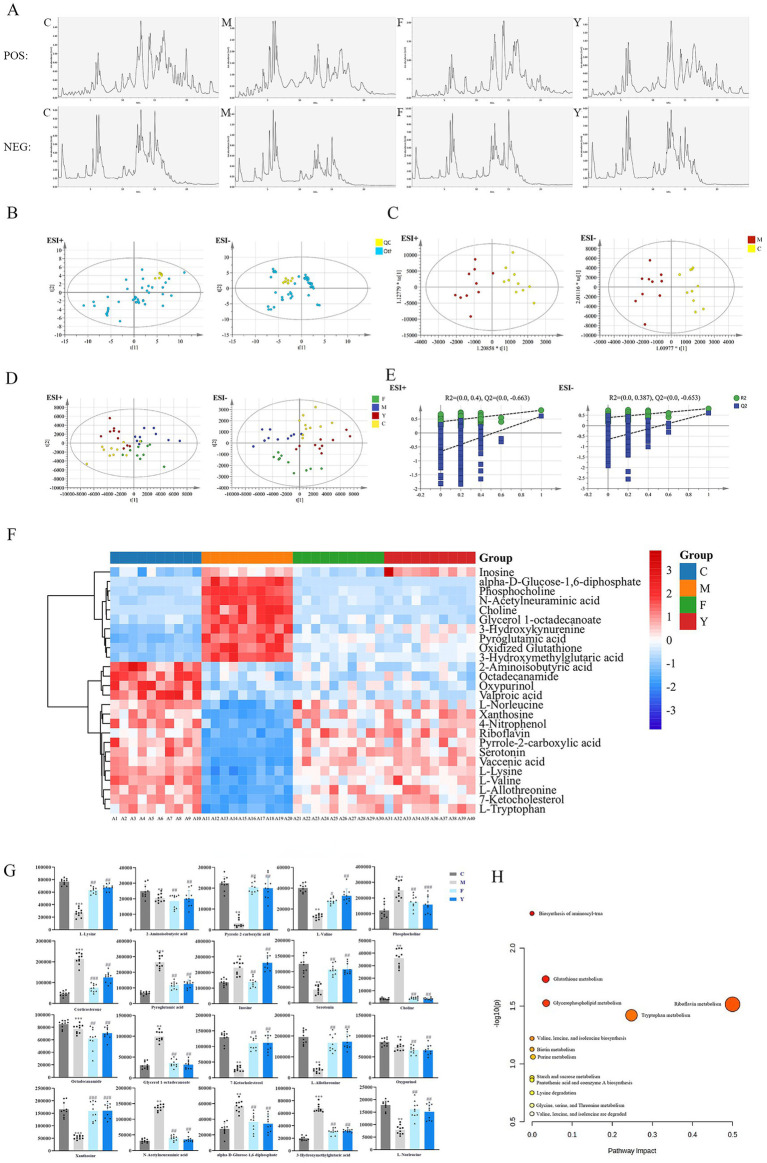
Effects of YHD on serum metabolic profile and differential metabolites of PCPA mice. **(A)** Total ion flow diagram of serum sample in positive group or in negative group. **(B)** PCA diagram of serum samples. **(C)** OPLS-DA diagram of serum sample. **(D)** PLS-DA diagram of serum sample (*n* = 10 per group). **(E)** OPLS-DA replacement test chart of serum sample. **(F)** Clustering heat map of biomarkers in serum samples (*n* = 10 per group). **(G)** Biomarker content map of serum samples (*n* = 10 per group). **(H)** Analysis of potential biomarker pathways in the serum of PCPA model mice. ^*^p < 0.01, ^***^p < 0.001 vs. C group; ^#^ *p* < 0.05, ^##^*p* < 0.01, ^###^*p* < 0.001 vs. M group **(H)** Analysis of potential differential metabolite pathways in serum of PCPA model ice.

In order to further explore the effect and mechanism of YHD on anxiety and depression-like behaviors in PCPA mice, we used LC–MS combined with MSDIAL software to characterize differential metabolites in serum, urine, and brain tissue, and assess the therapeutic potential of YHD. The total ion current diagrams of serum samples in positive and negative ion modes are shown below. (Figure 4A). PCA analysis showed strong clustering of QC samples, with retention time and relative peak area RSD values below 7%, indicating good reproducibility and stability of the method (Figure 4B). Supervised pattern recognition techniques, such as OPLS-DA and PLS-DA, were used to exhance sample separation. OPLS-DA analysis indicated clear separation between the C and M groups in both positive and negative ion modes (Figure 4C), suggesting the metabolomics of the model group was disordered and a metabolic profile shift in the treatment groups. Both the Y and F groups clustered more closely with the C group, indicating that YHD and fluoxetine treatments can improve the metabolomic disorder induced by PCPA model (Figure 4D).

In the OPLS-DA model permutation test (*n* = 200), the R2 and Q2 values from random permutations were smaller than the original values, and the Q2 intercept value was less than 0 (Figure 4E), validating the model without overfitting. The heatmap illustrates the effect of YHD on the serum metabolic profile of PCPA-induced mice and reveals the changes in differential metabolites. We identified 26 metabolites that were significantly altered between the C and M groups. Of these, 25 metabolites showed a tendency to revert toward normal levels following fluoxetine or YHD treatment (Table 3). Suggesting that YHD effectively improved metabolic disorders in PCPA model mice (Figure 4F). The relative contents of these 26 differential metabolites are shown in Figure 4G. Pathway enrichment analysis using MetaboAnalyst found 9 pathways related to differential metabolites, predominantly involving tryptophan metabolism and riboflavin metabolism (Figure 4H). These results indicate that YHD has a therapeutic effect on PCPA model mice, primarily through modulation of tryptophan and riboflavin metabolism.

**Table 3:** Comparison of serum metabonomic biomarkers among groups.

NO.	Formular	*m/z*	T_R_	Adducts	Identification	M group *VS* C group	Y group *VS* M group	F group *VS* M group
1	C_6_H_14_N_2_O_2_	147.1128	0.56	[M + H]^+^	L-Lysine	↓^***^	↑^##^	↑^##^
2	C_4_H_9_NO_2_	104.0707	0.68	[M + H]^+^	2-Aminoisobutyric acid	↓^**^	↓^##^	↓^##^
3	C_5_H_5_NO_2_	112.0491	0.69	[M + H]^+^	Pyrrole-2-carboxylic acid	↓^**^	↑^##^	↑^##^
4	C_5_H_11_NO_2_	118.0843	0.7	[M + H]^+^	L-Valine	↓^**^	↑^#^	↑^##^
5	C_5_H_15_NO_4_P	184.0747	0.72	[M + H]^+^	Phosphorylcholine	↑^***^	↑^##^	↑^##^
6	C_21_H_30_O_4_	346.4612	0.72	[M + H]^+^	Corticosterone	↑^***^	↓^###^	↓^##^
7	C_5_H_7_NO_3_	130.0505	0.73	[M + H]^+^	Pyroglutamic acid	↑^***^	↓^##^	↓^##^
8	C_10_H_12_N_4_O_5_	269.0883	0.77	[M + H]^+^	Inosine	↑^**^	↑^##^	↓^##^
9	C_10_H_12_N_2_O	177.0567	2.83	[M + H]^+^	Serotonin	↓^**^	↑^##^	↑^##^
10	C_5_H_14_NO	104.1082	13.03	[M + H]^+^	Choline	↑^**^	↓^##^	↓^##^
11	C_18_H_37_NO	284.2937	16.79	[M + H]^+^	Octadecanamide	↓^***^	↓^##^	↓^##^
12	C_21_H_42_O_4_	359.3148	17.16	[M + H]^+^	Glycerol 1-octadecanoate	↑^**^	↓^##^	↓^##^
13	C_27_H_44_O_2_	401.3427	17.67	[M + H]^+^	7-Ketocholesterol	↓^**^	↑^##^	↑^##^
14	C_4_H_9_NO_3_	118.0506	0.61	[M-H]^−^	L-Allothreonine	↓^**^	↑^##^	↑^##^
15	C_5_H_4_N_4_O_2_	151.0251	0.71	[M-H]^−^	Oxypurinol	↓^**^	↓^##^	↓^##^
16	C_10_H_12_N_4_O_6_	283.0664	0.84	[M-H]^−^	Xanthosine	↓^***^	↑^###^	↑^###^
17	C_11_H_19_NO_9_	308.0979	0.84	[M-H]^−^	N-Acetylneuraminic acid	↑^**^	↓^##^	↓^##^
18	C_6_H_14_O_12_P_2_	338.9887	0.87	[M-H]^−^	Alpha-D-Glucose 1,6-bisphosphate	↑^**^	↓^##^	↓^##^
19	C_6_H_10_O_5_	161.0469	0.91	[M-H]^−^	3-Hydroxymethylglutaric acid	↑^***^	↓^##^	↓^##^
20	C_6_H_13_NO_2_	130.0858	1.08	[M-H]^−^	L-Norleucine	↓^**^	↑^##^	↑^##^
21	C_11_H_12_N_2_O_2_	249.0876	2.27	[M-H]^−^	L-Tryptophan	↓^**^	↑^##^	↑^##^
22	C_10_H_12_N_2_O_4_	226.0799	2.59	[M-H]^−^	3-Hydroxykynurenine	↑^**^	↓^#^	↓^##^
23	C_17_H_20_N_4_O_6_	375.1294	3.17	[M-H]^−^	Riboflavin	↓^**^	↑^##^	↑^##^
24	C_6_H_5_NO_3_	138.0168	5.06	[M-H]^−^	4-Nitrophenol	↓^***^	↑^##^	↑^##^
25	C_8_H_16_O_2_	143.1068	9.28	[M-H]^−^	Valproic acid	↓^**^	↓^##^	↓^##^
26	C_18_H_34_O_2_	281.2489	16.49	[M-H]^−^	Vaccenic acid	↓^**^	↑^#^	↑^##^

^*^p < 0.05, ^**^p < 0.01 vs control group; ^#^ *p* < 0.05, ^##^*p* < 0.01, ^###^*p* < 0.001 vs model group.

Tables 2, 3 Comparison of serum metabonomic biomarkers among groups.

### 3.4 Effects of YHD on urinary metabolic profile and differential metabolites in PCPA model mice

**Figure 5:**
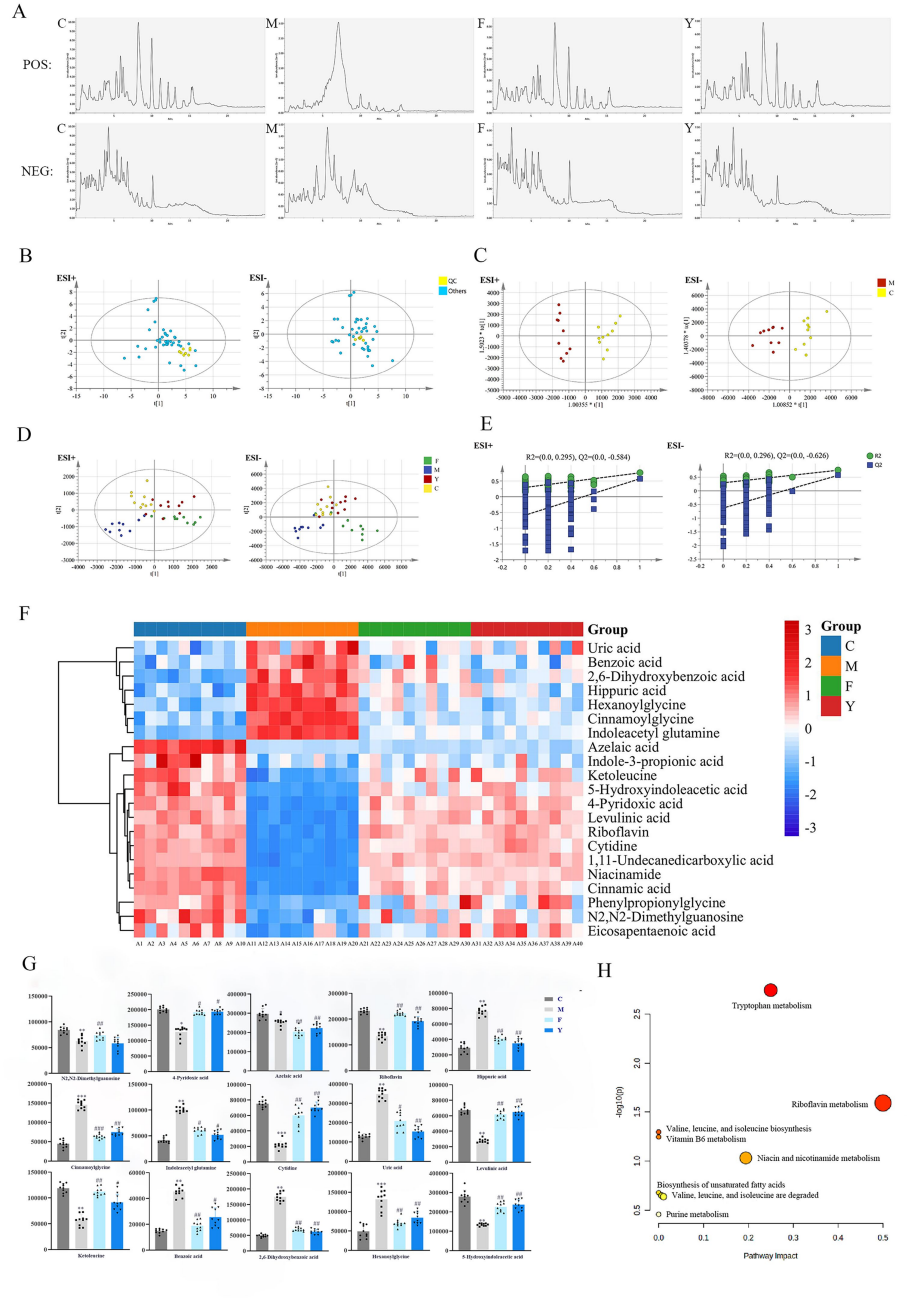
Effects of YHD on urine metabolic profile and differential metabolites of PCPA mice. **(A)** Total ion current diagram of urine sample in positive group or in negative group. **(B)** PCA diagram of urine samples. **(C)** OPLS-DA diagram of urine sample. **(D)** PLS-DA diagram of urine sample (*n* = 10 per group). **(E)** OPLS-DA replacement test chart of urine sample. **(F)** Cluster thermogram of biomarkers in urine samples (*n* = 10 per group). **(G)** Biomarker content map of urine sample (*n* = 10 per group). ^**^p < 0.01, ^***^p < 0.001 vs. C group; ^#^*p* < 0.05, ^##^ *p* < 0.01, ^###^ *p > p* < 0.001 vs. M group. **(H)** Analysis of urine potential biomarker pathway in PCPA model mice.

Next, we examined the urinary metabolic profiles and identified differential metabolites. The total ion current diagrams of urine samples in positive and negative ion modes are shown in Figure 5A. PCA analysis showed high clustering of QC samples, suggesting good reproducibility and stability (Figure 5B). OPLS-DA analysis demonstrated clear separation between the C and M groups, confirming successful replication of the PCPA model (Figure 5C). PLS-DA also indicated significant clustering and separation between groups (Figure 5D), suggesting a shift in the metabolic profile after treatment. Both YHD and fluoxetine groups resembled the C group more closely, highlighting the therapeutic effect of YHD on metabolic dysregulation in PCPA model mice.

The OPLS-DA model permutation test (n = 200) yielded valid results, with R2 and Q2 values smaller than those in the original model and a Q2 intercept value less than 0 (Figure 5E). Urinary differential metabolites were identified, revealing 21 metabolites, with 19 showing a tendency to revert following YHD or fluoxetine treatment (Table 4). Heatmap analysis demonstrated that the trends in the Y and F groups were similar to the C group, in contrast to the M group, indicating that YHD corrected metabolic disorders (Figure 5F). The relative contents of the differential metabolites are shown in Figure 5G.

**Table 4:** Comparison of urine metabonomics biomarkers among groups.

NO.	Formular	*m/z*	T_R_	Adducts	Identification	M group *VS* C group	Y group *VS* M group	F group *VS* M group
1	C_6_H_6_N_2_O	123.0541	0.79	[M + H]^+^	Niacinamide	↓^***^	↑^###^	↓^##^
2	C_8_H_9_NO_4_	184.0736	0.88	[M + H]^+^	4-Pyridoxic acid	↓^*^	↑^#^	↑^#^
3	C_12_H_17_N_5_O_5_	312.1275	1.19	[M + H]^+^	N2, N2-Dimethylguanosine	↓^**^	↓^##^	↑^##^
4	C_17_H_20_N_4_O_6_	379.1285	3.13	[M + H]^+^	Riboflavin	↓^**^	↑^##^	↑^##^
5	C_9_H_9_NO_3_	202.0467	4.2	[M + H]^+^	Hippuric acid	↑^**^	↓^##^	↓^##^
6	C_11_H_11_NO_3_	206.0809	5.05	[M + H]^+^	Cinnamoylglycine	↑^***^	↓^###^	↓^##^
7	C_15_H_17_N_3_O_4_	303.1072	5.1	[M + H]^+^	Indoleacetyl glutamine	↑^**^	↓^#^	↓^##^
8	C_9_H_13_N_3_O_5_	244.0929	7.39	[M + H]^+^	Cytidine	↓^***^	↑^##^	↑^##^
9	C_5_H_4_N_4_O_3_	167.0214	0.64	[M-H]^−^	Uric acid	↑^**^	↓^#^	↓^##^
10	C_5_H_8_O_3_	115.0411	1.12	[M-H]^−^	Levulinic acid	↓^**^	↑^##^	↑^##^
11	C_6_H_10_O_3_	129.0558	2.56	[M-H]^−^	Ketoleucine	↓^**^	↑^##^	↑^##^
12	C_7_H_6_O_2_	121.0297	2.74	[M-H]^−^	Benzoic acid	↑^**^	↓^##^	↓^#^
13	C_7_H_6_O_4_	153.019	3.07	[M-H]^−^	2,6-Dihydroxybenzoic acid	↑^**^	↓^##^	↓^##^
14	C_8_H_15_NO_3_	172.0978	3.94	[M-H]^−^	Hexanoylglycine	↑^***^	↓^##^	↓^##^
15	C_10_H_9_NO_3_	191.1899	4.46	[M-H]^−^	5-Hydroxyindoleacetic acid	↓^**^	↑^##^	↑^##^
16	C_11_H_13_NO_3_	206.0826	4.6	[M-H]^−^	Phenylpropionylglycine	↑^**^	↓^##^	↓^##^
17	C_9_H_16_O_4_	187.0966	5.82	[M-H]^−^	Azelaic acid	↓^*^	↓^#^	↓^##^
18	C_11_H_11_NO_2_	188.0726	6.74	[M-H]^−^	Indole-3-propionic acid	↓^**^	↑^##^	↑^##^
19	C_9_H_8_O_2_	147.0446	7.04	[M-H]^−^	Cinnamic acid	↓^**^	↑^##^	↑^##^
20	C_13_H_24_O_4_	243.1606	9.67	[M-H]^−^	1,11-Undecanedicarboxylic acid	↓^**^	↑^##^	↑^##^
21	C_20_H_30_O_2_	301.217	14.37	[M-H]^−^	Eicosapentaenoic acid	↓^**^	↑^##^	↑^##^

^*^p < 0.05, ^**^p < 0.01 vs control group; ^#^ *p* < 0.05, ^##^*p* < 0.01, ^###^*p* < 0.001 vs model group.

Pathway enrichment analysis identified 9 metabolic pathways affected by the treatment, further highlighting tryptophan and riboflavin metabolism as key pathways (Figure 5H). These findings further support the therapeutic effect of YHD in restoring metabolic balance in PCPA model mice.

Tables 2, 4 Comparison of urine metabonomics biomarkers among groups.

### 3.5 Effects of YHD on brain tissue metabolic profile and differential metabolites in PCPA model mice

**Figure 6:**
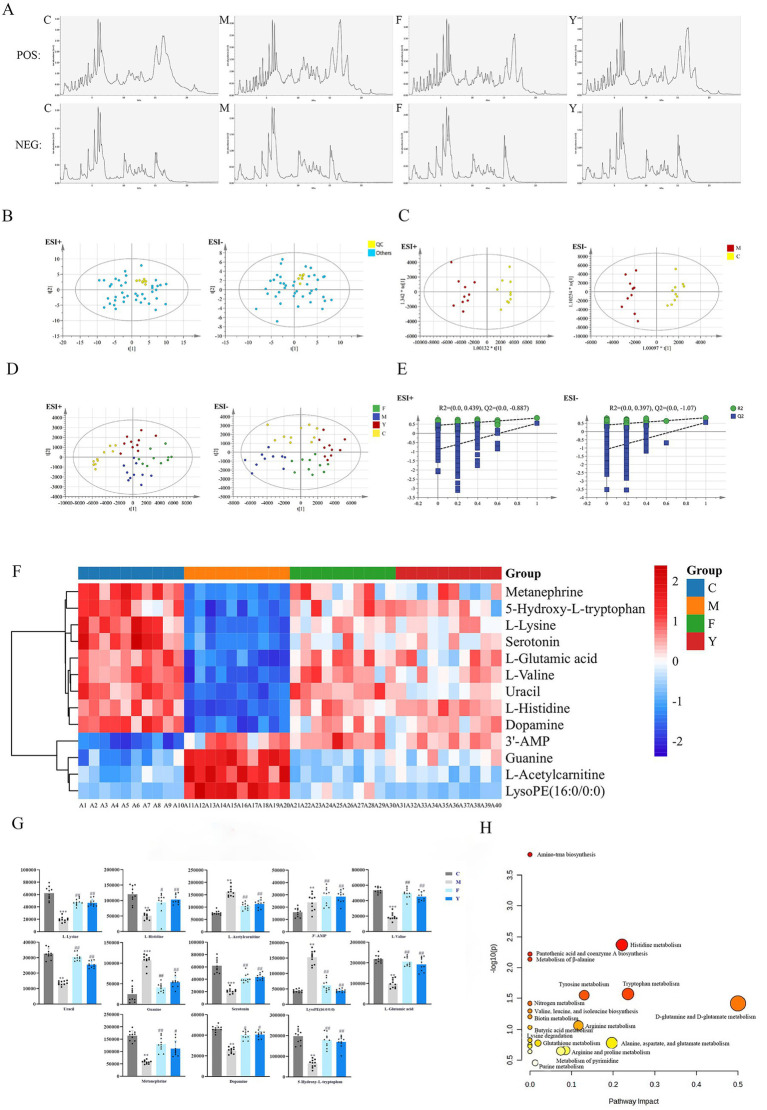
Effects of YHD on metabolic profile and differential metabolites in brain tissue of PCPA mice. **(A)** Total ion current diagram of brain tissue sample in positive group or in negative group. **(B)** PCA chart of brain tissue samples. **(C)** OPLS-DA diagram of brain tissue sample. **(D)** PLS-DA diagram of brain tissue samples (*n* = 10 per group). **(E)** OPLS-DA replacement inspection chart of brain tissue samples. **(F)** Cluster thermogram of biomarkers in brain tissue samples (*n* = 10 per group). **(G)** Biomarker content map of brain tissue samples (*n* = 10 per group). **(H)** Analysis of potential biomarker pathways in the brain tissue of PCPA model mice. ^**^p < 0.01; ^***^p < 0.001 vs. control group; ^#^*p* < 0.05; ^##^*p* < 0.01; ^###^*p* < 0.001 vs. model group>*p*. **(H)** Analysis of potential biomarker pathway in brain tissue of PCPA model mice.

Finally, we investigated the metabolic profiles and differential metabolites in brain tissue. The total ion current diagrams of brain tissue samples in positive and negative ion modes are shown below. (Figure 6A). PCA analysis indicated strong clustering of QC samples, confirming the stability of the analytical method (Figure 6B). OPLS-DA analysis demonstrated clear separation between the C and M groups (Figure 6C), confirming the successful establishment of the PCPA model. PLS-DA revealed significant clustering between groups, with the Y and F groups clustering more closely with the C group, suggesting that YHD improved the metabolic profiles of PCPA model mice (Figure 6D).

The OPLS-DA permutation test (*n* = 200) showed valid results with R2 and Q2 values lower than those from random permutations and a negative Q2 intercept (Figure 6E). The results in the table show that all 13 differential metabolites have a callback effect (Table 5). Heatmap analysis demonstrated that the trends in the Y and F groups were similar to the C group, opposite to the M group, indicating a correction in metabolic disorders following YHD treatment (Figure 6F). The relative contents of the differential metabolites are presented in Figure 6G.

**Table 5:** Comparison of metabonomic biomarkers of diencephalon in each group.

NO.	Formular	*m/z*	T_R_	Adducts	Identification	M group *VS* C group	Y group *VS* M group	F group *VS* M group
1	C_6_H_14_N_2_O_2_	147.1128	0.56	[M + H]^+^	L-Lysine	↓^***^	↑^##^	↑^##^
2	C_6_H_9_N_3_O_2_	156.0759	0.57	[M + H]^+^	L-Histidine	↓^**^	↑^#^	↑^##^
3	C_9_H_17_NO_4_	204.1227	0.69	[M + H]^+^	L-Acetylcarnitine	↑^**^	↓^##^	↓^##^
4	C_10_H_14_N_5_O_7_P	348.0713	0.69	[M + H]^+^	3’-AMP	↑^**^	↑^##^	↑^##^
5	C_5_H_11_NO_2_	118.0843	0.7	[M + H]^+^	L-Valine	↓^***^	↑^###^	↑^##^
6	C_4_H_4_N_2_O_2_	113.033	0.74	[M + H]^+^	Uracil	↓^**^	↑^##^	↑^##^
7	C_5_H_5_N_5_O	152.0566	0.76	[M + H]^+^	Guanine	↑^***^	↓^###^	↓^##^
8	C_10_H_12_N_2_O	177.0567	2.83	[M + H]^+^	Serotonin	↓^***^	↑^##^	↑^##^
9	C_21_H_44_NO_7_P	454.2909	12.98	[M + H]^+^	LysoPE (16:0/0:0)	↑^**^	↓^##^	↓^##^
10	C_5_H_9_NO_4_	148.0629	0.71	[M-H]^−^	L-Glutamic acid	↓^**^	↑^##^	↑^##^
11	C_10_H_15_NO_3_	198.1136	4.03	[M-H]^−^	Metanephrine	↓^**^	↑^##^	↑^##^
12	C_8_H_11_NO_2_	307.1663	4.22	[M-H]^−^	Dopamine	↓^**^	↑^#^	↑^#^
13	C_11_H_12_N_2_O_3_	221.2913	5.31	[M-H]^−^	5-Hydroxy-L-tryptophan	↓^**^	↑^##^	↑^##^

^*^ *p* < 0.05, ^**^ *p* < 0.01 vs control group; ^#^ *p* < 0.05, ^##^ *p* < 0.01, ^###^ *p* < 0.001 vs model group.

Pathway enrichment analysis revealed 21 key metabolic pathways, including tryptophan metabolism, histidine metabolism, glutamate metabolism, and tyrosine metabolism (Figure 6H). These findings suggest that YHD exerts a therapeutic effect on PCPA model mice, primarily through regulation of these metabolic pathways.

Tables 2, 5 Comparison of metabonomic biomarkers of diencephalon in each group.

### 3.6 Network pharmacology results of YHD in PCPA model mice

**Figure 7:**
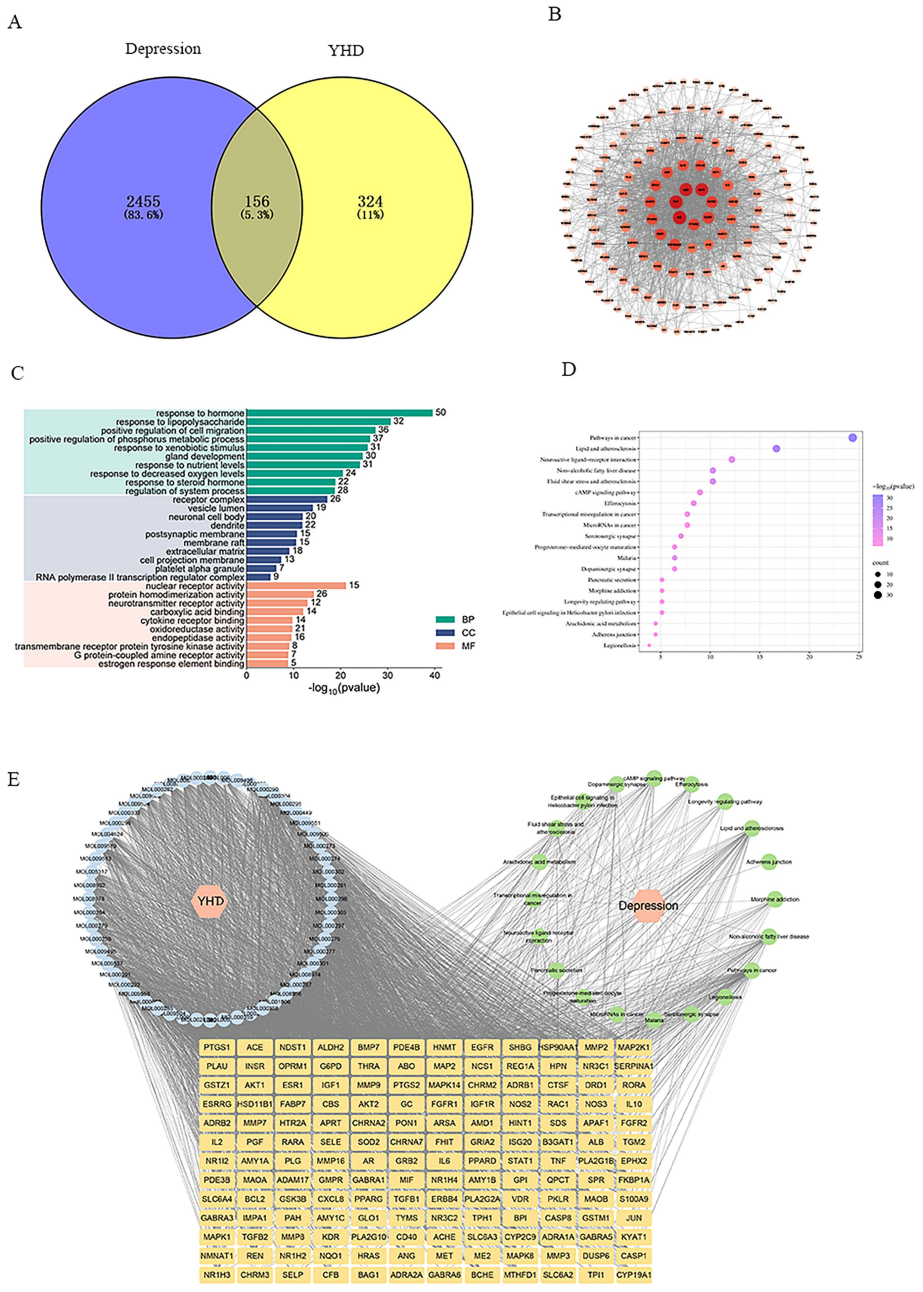
Results of network pharmacology of YHD in PCPA mice. **(A)** Venn diagram for the common targets of YHD and depression. **(B)** PPI network diagram of common targets of YHD and depression. **(C)** GO enrichment analysis of common targets of YHD and depression. **(D)** KEGG pathway enrichment analysis of common targets of YHD and depression. **(E)** Network diagram of YHD-brain–blood-absorbed ingredients–depression-targets-pathway.

A total of 480 active ingredient targets of YHD were collected from the TCMSP, SwissTargetPrediction, and PharmMapper databases, and 2,611 depression-related targets were obtained from DisGeNET, OMIM, and GeneCards. A Venn diagram revealed 156 intersection targets between YHD and depression (Figure 7A). These intersecting targets were input into the STRING platform to predict protein–protein interactions, which were visualized and analyzed in Cytoscape to generate a protein–protein interaction (PPI) network comprising 150 nodes and 1,628 edges. Degree represents the number of connections between a node and other nodes in the network. The more connections, the greater the degree value, and the more important the node is in this network. The node size indicates the Degree value, with larger and darker nodes representing higher Degree values. The PPI network identified the top seven intersecting targets with the highest Degree values: AKT1, ALB, TNF, IL-6, PTGS2, EGFR, and TGF-β1 (Figure 7B).

GO functional enrichment analysis revealed that YHD treatment of depression may regulate biological processes such as response to hormones and lipopolysaccharides, act on cellular components like vesicle lumen and receptor complexes, and influence molecular functions such as nuclear receptor and neurotransmitter receptor activity. KEGG pathway enrichment analysis indicated that these targets were mainly involved in pathways such as neuroactive ligand-receptor interactions and dopaminergic synapses (Figures 7C,D).

In summary, YHD may improve depressive symptoms by modulating hormonal responses and LPS responses affecting key pathways such as neuroactive ligand-receptor interactions and dopaminergic synapses, as demonstrated in the “YHD component-depression-target-pathway” network (Figure 7E), where multiple active compounds in YHD regulate multiple targets to alleviate depression.

## 4 Discussion

Depression has now become an increasingly serious public health issue, imposing a heavy burden on individuals and society (Li et al., 2022). The main cause is the interaction between genes and environment, leading to abnormal changes in genes and signaling pathways.

PCPA, a selective inhibitor of tryptophan hydroxylase that limits the synthesis of 5-HT, is commonly used to mimic depression models, which is used in the research of antidepressant drug screening and the pathophysiological mechanism (Hong et al., 2020).

The behavioral characteristics of depression include anhedonia, slowed thinking, and low mood, etc. (Liu et al., 2018). In animal experiments, SPT is often used to assess the defect of pleasure (Verharen et al., 2023). FST, TST, and OFT evaluate the autonomy, exploratory behavior, and tension of animals in a new environment, and are used to observe the autonomous movement ability (Nadeau et al., 2022). In this study, behavioral tests indicated that YHD and fluoxetine have similar efficacy in changing depressive behaviors. In histological analysis, it was shown that YHD has a protective effect on neuronal damage induced by PCPA, and can exert the effect of improving neuronal morphology and the integrity of Nissl bodies. The cell structure of mice treated with YHD was enhanced, the neuronal density increased, and Nissl bodies were retained. YHD improved the depressive-like and despair-like behaviors of PCPA mice. Therefore, YHD shows potential as an efficient and safe antidepressant drug. Based on this, using metabolomics methods, differential metabolite analysis was conducted on serum, urine and brain tissue to clarify the direction of YHD’s protective mechanism for the PCPA model mice.

The experimental results indicate that YHD may improve behavioral phenomena by regulating certain differential metabolites. Specifically, serotonin, dopamine, 5-hydroxy-L-tryptophan, and 5-hydroxyindoleacetic acid, as core neurotransmitters or their precursors, have abnormal levels, which directly lead to depressive symptoms such as a lack of pleasure and motivation. These are the key targets for anti-depression intervention (Meltzer, 1989). The supplementation of 5-hydroxy-L-tryptophan can significantly increase the sucrose preference rate of patients with depression and shorten the immobility time in the forced swimming test (D'elia et al., 1978). The baseline release levels of serotonin and dopamine in the prefrontal cortex and ventral hippocampus of the depression model mice were decreased, which was directly related to the prolonged immobility in the forced swimming and tail suspension tests as well as the reduced sucrose preference (Romano et al., 2014).

Furthermore, the results of serum metabolomics indicated that the expression levels of various amino acids decreased in the M group mice, including tryptophan, valine, lysine, etc. This might be due to the fact that the model mice lost their sense of pleasure, reduced food intake, and experienced weight loss, thereby slowing down the body’s basal metabolism and causing metabolic disorders of amino acids; the results of urine metabolomics showed that the expression level of creatine acid in the urine of the M group mice increased. As a metabolite of benzoic acid shared by intestinal microorganisms and mammals, its generation in the mammalian body mainly depends on the intestinal microbiota (Xu et al., 2024). The results of brain tissue metabolomics indicate that they are consistent with the classic theory of imbalance in monoamine neurotransmitters. The differential metabolites in group M mice include neurotransmitters such as 5-HT, dopamine (DA), and norepinephrine (NE), and their expression levels have decreased. After taking YHD, the above differential metabolites all returned to normal, suggesting that YHD may exert therapeutic effects through multiple pathways, such as improving amino acid metabolism, inhibiting the overexcitation of the hypothalamic–pituitary–adrenal axis, regulating the intestinal flora, and promoting the expression of neurotransmitters.

Based on the results of KEGG enrichment analysis, it was found that the metabolomics of urine, serum and brain tissue all involve tryptophan metabolism and riboflavin metabolism. The identified relevant differential metabolites in the metabolic pathways indicate that this might be the key mechanism by which YHD exerts its therapeutic effect. Tryptophan is converted into 5-hydroxytryptamine (5-HTP) by tryptophan hydroxylase (TPH), and then deoxygenated to form 5-hydroxytryptamine (Ameisen et al., 1989; Xie et al., 2023). Furthermore, the regulation of tryptophan metabolism also involves converting tryptophan into other metabolites, such as quinonic acid, which is closely related to neuroinflammation and immune responses (Cervenka et al., 2017; Platten et al., 2019; Baumgartner et al., 2019), and can regulate the immune response by activating the aryl hydrocarbon receptor (AhR), thereby alleviating inflammatory effects (Zang et al., 2018). Currently, increasing the content of tryptophan is a potential method for treating depression (Kałużna-Czaplińska et al., 2019). In addition, the riboflavin metabolic pathway is also one of the pathways with a relatively high correlation. Riboflavin belongs to the vitamin B group (Wu et al., 2023), and B vitamins have been proven to be able to modify depressive symptoms through various mechanisms, such as reducing oxidative stress, inhibiting neuroinflammation, and altering short-chain fatty acids or neurotransmitters (Śliwiński and Gawlik-Kotelnicka, 2024; Qureshi et al., 2011). Therefore, YHD may exert an antidepressant effect by regulating tryptophan metabolism and riboflavin metabolism, thereby inhibiting neuroinflammation.

In order to further predict the potential targets of YHD’s antidepressant effect, we used network pharmacology to find 156 cross-targets between YHD and genes related to depression. Among them, AKT1, ALB, TNF, IL-6, PTGS2, EGFR, and TGF-β1 are core targets closely related to depression and play a key role in the inflammation-neuroplasticity-emotional regulation pathway. Studies have shown that in depression animal models, there is a significant inflammatory response in the brain and peripheral blood (Husain et al., 2017). Clinical research has found that in patients with depression, various inflammatory-related indicators (such as TNF-*α* and IL-6) are usually elevated in the body, which may affect the function of the nervous system, inhibit neuroplasticity, promote nerve cell damage, and ultimately aggravate depressive symptoms (Ghaffari Darab et al., 2020). AKT1 is the core node of the PI3K/AKT signaling pathway. It not only regulates cell survival and anti-apoptosis processes, but also participates in neuronal plasticity and stress adaptation by influencing the CREB/BDNF pathway (Mathew et al., 2008). ALB enhances its bioavailability by combining with tryptophan to promote serotonin synthesis, and also exhibits antioxidant and anti-inflammatory effects. TNF-α weakens the neural plasticity of the hippocampus and prefrontal cortex by activating the NF-κB pathway, inducing neuroinflammation, and inhibiting the expression of brain-derived neurotrophic factor (BDNF), thereby exacerbating depression. Moreover, TNF-α antagonists have been proven to effectively alleviate depressive-like behavioral symptoms in patients and animal models (Babri et al., 2014). TNF-α can weaken the neural plasticity of the hippocampus and prefrontal cortex by activating the NF-κB pathway, inducing neuroinflammation, and inhibiting the expression of brain-derived neurotrophic factor (BDNF), thereby exacerbating depression. IL-6, as a typical pro-inflammatory cytokine, numerous clinical studies have shown that the serum IL-6 level in patients with depression is significantly elevated, and it is positively correlated with persistent attention impairment. This suggests that IL-6 may be involved in the occurrence of depression-related cognitive dysfunction by disrupting the excitability and synaptic plasticity of the prefrontal–limbic system network (Kelly et al., 2021). Studies have shown that IL-6 can bind to its receptor IL-6R, activating the JAK-STAT3 pathway, which leads to the release of more pro-inflammatory factors (such as TNF-α and IL-1β) by glial cells, thereby exacerbating depression (Rossetti et al., 2022).

Therefore, with AKT1, TNF, and IL-6 as representative core targets, the peripheral inflammatory response is closely linked to central neural plasticity, emotional regulation, and cognitive control.

Based on the above role of metabolomics in depression, and the network pharmacology analysis of core targets such as AKT1, TNF, and IL-6 and their regulatory pathways, we further analyzed the combination of the two and constructed an “abnormal metabolism - molecular mechanism” correlation framework to explore the interrelationships between key metabolites and core targets. Metabolomics studies have shown that tryptophan and riboflavin are the main metabolic pathways for the antidepressant effect of YHD. Tryptophan can be metabolized through the following three pathways: The 5-hydroxytryptamine pathway: Tryptophan is converted into 5-hydroxytryptamine (5-HTP) by tryptophan hydroxylase (TPH), and then dehydrated to 5-HT (Ameisen et al., 1989; Xie et al., 2023); The kynurenine pathway: Tryptophan is catalytically hydrolyzed by indoleamine 2,3-dioxygenase to generate kynurenine; The indole pathway: Tryptophan is catalyzed by tryptophanase to generate indole and its derivatives. 5-hydroxytryptamine directly regulates the generation of 5-hydroxytryptamine, inflammatory factors TNF, IL-6, etc. can induce the activation of indoleamine 2,3-dioxygenase, promoting the diversion of tryptophan to kynurenine, thereby reducing the generation of 5-HT and exacerbating depression; Indole derivatives can regulate the balance of pro-inflammatory/anti-inflammatory factors and indirectly affect the AKT1/PI3K pathways involved in the antidepressant process (Öztürk et al., 2021).

In the metabolism of riboflavin, riboflavin is metabolized by riboflavin kinase (RFK) to produce flavin mononucleotide (FMN), which, under the action of FAD synthase, generates the final product flavin adenine dinucleotide (FAD). The FMN can inhibit the expression of RFK by regulating lysine-specific methyltransferase 2B (KMT2B), thereby blocking the release of pro-inflammatory factors TNF and IL-6. Moreover, riboflavin can enhance the activity of antioxidant enzymes and reduce oxidative stress, indirectly regulating the AKT1-mediated PI3K/AKT signaling pathway (Maes et al., 2011).

YHD’s antidepressant effects are likely a result of the synergistic actions of its multiple components, which target various key signaling pathways and molecular targets. Based on network pharmacology analysis, the primary targets of YHD include AKT1, TNF, IL-6, and EGFR, which play crucial roles in neuroplasticity, immune response, and neurotransmitter metabolism. Specifically, YHD may act by modulating the AKT1/PI3K pathway, restoring neuroplasticity, and improving neuronal function, thereby alleviating depressive symptoms. Moreover, YHD exerts anti-inflammatory effects by suppressing pro-inflammatory cytokines such as TNF and IL-6, further contributing to its antidepressant effects.

Unlike traditional antidepressants such as fluoxetine, which act through a single mechanism, YHD as a compound traditional Chinese medicine (TCM) exhibits a multidimensional therapeutic mechanism. YHD not only regulates neurotransmitter balance (such as through tryptophan and riboflavin metabolism) to improve neuronal function but also works by suppressing neuroinflammation, modulating immune responses, and enhancing neuroplasticity. This comprehensive mechanism of action suggests that YHD may be superior in its ability to address multiple pathological mechanisms of depression. Furthermore, given its potential for lower side effects and better long-term tolerability, YHD may complement existing medications like fluoxetine, particularly in cases where fluoxetine’s efficacy is limited or resistance develops. In conclusion, YHD, with its multi-target action and reduced side effects, holds significant potential as a promising candidate for future antidepressant therapies.

### 4.1 Limitation

First, although this study demonstrates the antidepressant effects of YHD in the PCPA-induced 5-HT depletion model, depression is a multifactorial disorder that may involve additional mechanisms such as neuroinflammation and chronic stress. Future studies should replicate these findings in other depression models, such as chronic stress, inflammation, and learned helplessness, to confirm the broader therapeutic potential of YHD.

Second, this study has a methodological limitation in that histological analysis was focused on the hippocampus, while ELISA and metabolomics were conducted using whole-brain hemisphere homogenates. While whole-brain homogenates provide valuable insights into global neurochemical and metabolic changes, they do not allow for detailed regional analysis. Future studies will focus on more targeted sampling of specific brain regions, such as the hippocampus and prefrontal cortex, to achieve a better understanding of the localized effects of YHD.

## 5 Conclusion

In conclusion, this study demonstrates the potential of YHD, a traditional Chinese medicine formula, in alleviating depressive symptoms through its multi-target, multi-pathway mechanisms. YHD effectively improved behavioral parameters and reversed neuronal damage in the PCPA-induced depression model, with its effects comparable to fluoxetine. The modulation of the serotonergic system, particularly through the restoration of brain 5-HT levels, along with the normalization of tryptophan metabolism, highlights its role in neurotransmitter balance and neuroprotection. Network pharmacology further identified key targets involved in inflammation, neuroprotection, and neurotransmission, supporting the multi-faceted nature of YHD’s antidepressant effects. These findings provide a solid foundation for further research into the molecular mechanisms underlying YHD’s action, offering the potential for more personalized and effective treatment strategies for depression. Future studies should focus on exploring the intricate relationships between YHD’s metabolic, neurochemical, and inflammatory effects to fully elucidate its therapeutic potential.

## Data Availability

The original contributions presented in the study are included in the article/supplementary material, further inquiries can be directed to the corresponding author.

## Footnotes

1
https://mona.fiehnlab.ucdavis.edu/downloads

2
https://www.metaboanalyst.ca
